# Prevalence of and Risk factors associated with hypertension: a community based- cross sectional study in Ndorwa West Health Sub District, Kabale district, southwestern Uganda

**DOI:** 10.21203/rs.3.rs-4579650/v1

**Published:** 2024-07-02

**Authors:** John Bosco Munezero Tamu, Valence Mfitumukiza, Christiana Nkiru Okafor, Immaculate Mandera, Jane Kabami, Edward Bwengye Arineitwe, Lydia Namuyibwa, Herbert Izo, Everd Baikaitwoha, Uchenna Prosper Okonkwo

**Affiliations:** Kabale University School of Medicine; Kabale University School of Medicine; Kabale University School of Medicine; Kabale University School of Medicine; Kabale University School of Medicine; Edward Bwengye Arineitwe. Kabale local government; Lydia Namuyibwa, Kabale University School of Medicine; Kabale University School of Medicine; Everd Baikaitwoha. Maniple. School of medicine, Kabale University; Nnamdi Azikiwe University

**Keywords:** Prevalence, Hypertension, Risk factors, Community, Uganda

## Abstract

**Background:**

Globally, one billion people have hypertension (HT), it kills 9.4 million people annually. Prevalence is higher in developed countries and is rapidly rising in developing countries, and approximately 31.5% of Ugandans have HT.

**Objective:**

This study aimed to determine the prevalence of and risk factors associated with HT among adults aged 25–65 years in the Ndorwa West HSD, Kabale District.

**Methods:**

A community-based cross-sectional survey was conducted with 381 adults aged 25–65 years in Ndorwa West HSD, using a modified WHO STEPwise approach to chronic disease risk factor surveillance. Chi-square tests with 95% Confidence Intervals (CI) and p-values less than 0.05, were used to assess the association between hypertension and associated factors. Odds Ratios (OR) with their corresponding 95% confidence intervals (95% CI) estimated the risk.

**Results:**

The prevalence of HT and pre-HT in Ndorwa HSD was 28.3% and 45.7%, respectively. 61.8% (n = 243) were females and 36.1% (n = 142) were males with a mean age of 48.18 years and standard deviation of 11.5 years. The mean Body Mass Index (BMI) was 25.92 kg/m^2^ and the standard deviation was 3.69 kg/m^2^. Only Age (p = 0.010, OR = 1.81(1.14–2.87) and level of education (p = 0.04) were significantly associated with hypertension. age ≥ 45years increased the likelihood of developing HT by 0.81 times. Behavioral factors associated with HT included awareness of the BP status (p = 0.010, OR = 0.53(CI: 0.32–0.87),use of fats/oil for cooking (p = 0.02, OR = 1.73 (CI: 1.09–2.75)), reduced salt intake (p = 0.001, OR = 0.075(CI:0.01–0.55)), and overweight and obesity (BMI) level ( p = 0.010, OR = 1.77 (CI 1.12–2.80)). BMI ≥ 25kg/m^2^ increased the likelihood of developing HT by 0.77 times.

**Conclusion:**

The prevalence of HT and pre-HT in this rapidly transitioning rural–urban population was high. The risk of CVDs is about 16 folds higher among pre-HT compared to no HT and doubles for every 10-mmHg increase in BP. Hence, the considerable risk and burden of HT and related CVDs that require a dire need to adopt strategies to prevent and control hypertension based on the identified associated risk factors in Ndorwa HSD.

## Introduction

Globally, Hypertension (HT) is one of the most common non-communicable diseases (NCDs) and a major contributor to approximately three-fourths of mortality from cardiovascular disease and stroke[1]. In 2015 alone, it was responsible for over 212 million disability-adjusted life-years (DALYs) globally, indicating a 40% rise since 1990 [2]. HT is defined as abnormally high arterial blood pressure due to a persistently raised force of blood pushing against the walls of arteries as the heart pumps it [3]. It is a silent and invisible killer that rarely causes symptoms [4].

There is increasing evidence of the incidence of NCDs in low-income countries in sub-Saharan Africa (SSA), and this increase is attributed to interrelated changes in socio-demographic determinants influenced by globalization [5]. A number of studies also indicate that the adoption of a Western behavioral lifestyle is linked with an increased prevalence of obesity, alcohol and tobacco consumption, physical inactivity, and transition from rural agricultural work to more globalized urban lifestyles [6].

Recent studies in Uganda have highlighted variations in the prevalence of hypertension between regions and in demographic groups between men and women [7, 8]. In 2018, the national prevalence of hypertension among adults in Uganda was 31.5% ^2^. It was higher among males (34.2%) than among females (30.2%), and 90% of individuals with HT were reported to be unaware of it.

The prevalence of HT in Uganda was highest in the western region of Uganda, including Kabale district at 26.3% of the total population [2]. However, the prevalence of HT in the Ndorwa West Health Sub-District (HSD) remains unknown. Moreover, there are high trends of HT prevalence in similar rural communities due to socio-demographic and behavioral transition [9]. Hence, a community-based, cross-sectional study among adults aged 25–65 years done and assessed the prevalence and factors associated with HT in the Ndorwa West HSD and associated factors, since there is unknown amidst evidence of high prevalence in similar rural areas. This study intends to fill the gap by determining the prevalence of HP and Pre-HBP in Ndorwa west Health Sub-Distric.

## Methods

Study Design, Setting, and Participants: This study used a community-based, cross-sectional design that assessed the prevalence of and risk factors associated with hypertension in Ndorwa HSD. The setting comprised the sub-counties Kitumba, Kamuganguzi, Katuna, Ryakirimira, Rubaya, and Butanda. The Ndorwa West HSD has a population of approximately 102,500 people[10] is bordered by Kabale Municipality to the north, Ndorwa East HSD to the east, Republic of Rwanda to the south, and Rubanda District to the west. The Katuna Town Council, the main town, lies on the border of Rwanda and is approximately 470 km (300 mi) by road, southwest of Kampala, the capital of Uganda, and approximately 25 km (16 mi) south of Kabale District. The main activities in Katuna, Ryakirimira, and Kitumba are cross-border trade and retail businesses. This has led to a shift from traditional manual farming, which is both physically demanding and intense. People’s settlements have also progressively moved downhill from the hilltops to the lowlands where modern transport is accessible, which could reduce physical exertion. The WHO STEPwise approach according [11] was modified to suit the Ndorwa context to provide a simple, standardized method for collecting and analyzing associated risk factors among adults aged 25–65 years. The WHO STEP-wise approach was household-based, interviewer-administered, and collected data in two steps: step 1 used a questionnaire for demographic and lifestyle/behavioral data, and step 2 involved physical measurements of BP, height, and weight, which were used for BMI calculations.

### Sample size and sampling procedure:

The sample size of 384 was determined using the Israel Glen formula^12^. It assumed the prevalence of hypertension to be 50% because it was unknown, permissible error of 5%, and the level of confidence of 95%. Additional non-response rate of 10% (39) was included to provide a final sample size of 423 participants.

A 3-stage sampling procedure was used: Stage 1. Selection of parishes from six counties using systematic sampling. Stage 2 involved the selection of households from each parish using simple random sampling. Stage 3. Involved in the convenient selection of a member of the target age group from each of the selected households. When the sampled household had more than one eligible participant available, one member was randomly chosen using the lottery method. In the case of the non-availability of eligible persons in a selected household, an adjacent household was considered.

#### Data Collection.

The selected households were accessed with the help of the Village Health team (VHT). Face-to-face interviews based on the WHO STEP-wise questionnaire for NCDs surveillance tailored to the study setting were conducted, and data regarding sociodemographic variables, lifestyle, and behavioral factors were collected by a Registered Nurse trained as a research assistant.

Physical measurements of participants’ (blood pressure, height, and weight were obtained without heavy outdoor clothing after the interview. Height was measured to the nearest millimeter using an anthropometric rod. Weight was measured using a pre-standardized digital body weighing scale. BMI was calculated using the formula [weight in kilograms divided by height in meters squared] and classified as Underweight-BMI < 18.5 kg/m2, normal weight- BMI 18.5–24.9 kg/m2.,Overweight- BMI 25.0–29.9 kg/m2 and Obese- BMI > 30.0 kg/m^2^.

Blood pressure was measured using a mercury sphygmomanometer with the participants in a sitting position after resting for at least five minutes. Three BP measurements on a single visit were taken at least one minute apart and the average was recorded. Male and female research assistants measured the BP of male and female participants, respectively, to avoid opposite-sex anxiety. BP readings were classified as **Normotension**, with an average systolic BP of 80–120 mmHg or diastolic BP of 60–79 mmHg; **Pre-hypertension**, systolic BP = 121–139 mmHg or diastolic BP = 80–89 mmHg; **Hypertension** where the average systolic BP was ≥ 140 mmHg and or diastolic BP was ≥ 90 mmHg; and **hypotension**, defined as an average systolic BP < 79 mmHg and or diastolic BP of < 59 mmHg.

## Data Analysis

The analysis was performed using Statistical Package for the Social Sciences software (IBM SPSS version 20). Descriptive statistics were summarized and presented using simple frequency distribution tables. The prevalence of hypertension was determined as the percentage of participants classified as hypertensive with all study participants as the denominator. Prevalence was then standardized by age and sex using the method according to a previous study [13]. Chi-square tests (p < p-values less than 0.05) were used to assess the association between hypertension and associated risk factors. Odds Ratios (OR) with their corresponding 95% confidence intervals (95% CI) provided the risk estimates of the associated risk factors.

Ethical considerations: Ethical approval was obtained from the Research Ethics Committee of Mbarara University of Science and Technology (MUST) Research Ethics Committee (REC). All participants provided written informed consent before voluntarily participating in the study. Participants with elevated blood pressure and no adherence to treatment were counselled and referred to their nearest health facility for further action and follow-up. VHTs assisted by home access were requested to leave private interviews and blood pressure measurements. The questionnaires were coded to ensure the non-identification of the participants. The completed questionnaires were immediately kept under a lockable cupboard that was only accessible to the principal investigator until data entry for analysis and report publication. The entire community was acknowledged rather than the individual participants.

## Results

Out of the 423 participants, 385 participants had complete data, giving a response rate of 91%.

### Socio-demographic and behavioral factors

The majority (61.8%) were females and the mean age was 48.18, SD 11.5 years. The mean BMI was 25.92 kg/m^2,^ SD 2.69 kg/m^2^). Most of the participants were non-smokers as only 13.2%( n = 51) reported smoking. However, 57.4% reported alcohol consumption. Only one third (39.2%) reported participation in activities that required exertion e.g. walking uphill or bicycling, while another one third reported fats/oil use in their diet.

### Prevalence of hypertension

Age was significantly associated with HT (p value = 0.010, OR = 1.81(1.14–2.87). Age > 45years increased the likelihood of developing HT by 0.81 times (Table 2).

### Socio demographic and behavioral factors associated with HT

Only Age category and level of education were significantly associated sociodemographic factors with HT.

Behavioral factors associated with HT included awareness of the BP status (p = 0.010, OR = 0.53(CI: 0.32–0.87), use of fats/oil for cooking (p = 0.02, OR = 1.73 (CI: 1.09–2.75)), reduced salt intake (p = 0.001, OR = 0.075(CI:0.01–0.55)), and overweight and obesity (BMI) level ( p = 0.010, OR = 1.77 (CI 1.12–2.80)). BMI ≥ 25kg/m^2^ increased the likelihood of developing HT by 0.77 times.

## Discussion

The findings of the prevalence of hypertension and associated risk factors among adults aged 25–65 years from Ndorwa west HSD community based study, revealed a prevalence of hypertension of 28.3%. This prevalence is slightly higher compared to Western Uganda region and other Ugandan rural areas, which were 26.3% and 25.8%, respectively [8]. It is also higher than prevalence in a similar setting study in Burkina Faso, which reported 18% [14]. the prevalence however was lower compared in a study conducted in similar setting in Sudan, which reported a 40.8% prevalence^15^.

The prevalence of hypertension in Ndorwa HSD however is consistent with findings from two community based studies in Ethiopia, which reported 28.3% and 27.4% [16, 17]. It also concurs with that reported in rural areas of the central region of Uganda of 28.5%^8^.

The high prevalence in this community based study is most likely due to an increase in adoption of modern lifestyles characterized by less exercise and high fat diet, which could be linked with an increased prevalence of obesity since less than half of the participants (44.9%) had Normal BMI > 24.5kg/m^2^. In this study, BMI was significantly associated with hypertension (p = 0.013). BMI > 24.9 kg/m^2^ compared to < 25 kg/M^2^ increased the odds of developing hypertension by 0.79. Therefore, Ndorwa HSD should not delay initiating interventions that improve physical activity and a healthy diet through community-based strategies that incorporate informational, behavioral, and social policy-making.

Although females had a higher pre-HT and HT prevalence than men, sex was not found to be significantly associated with HT. However, the observed difference can be explained by the fact that females engage in less physical activity at home, which makes it easier for them to accumulate fat and become more obese, and hence more prone to developing hypertension^18^.

The prevalence of pre-HT was 47.5%, which was higher than 30.6% reported by meta- analysis studies conducted in Middle East and North Africa [19], and in Nepal [20].

The high prevalence of pre-HT represents an increased risk of developing hypertension and other associated cardiovascular diseases. Pre-HT progresses to clinical hypertension at a rate of 19% over four years [21, 22]. Therefore, it is important to note that a high prevalence of pre-HT reflects an emerging public health concern because of progression to hypertension and other associated CVDs. This calls for the need to strengthen Hypertension and other NCD prevention programs to prevent the progression to hypertension and reduce related CVDs.

Behavioral factors associated with HT included awareness of BP status, reduced salt intake, use of fat/oil for cooking, and overweight and obesity.

The study noted that 89.5% had ever had their BP measured at least once, and 87.8% were aware of their blood pressure status contrary to^2^ finding of 90% not aware of their BP status. This means that remaining 12.2% are still unknowingly at risk of HT and its complications, since it is a silent killer [4]. Those with a BP status are more likely to take up HT prevention compared to those who are not and have reduced odds of developing HT.

Reduced salt intake was associated with lower odds of developing HT. Among those who reported high salt intake, one out of three were hypertensive and approximately one out of two were pre-hypertensive.

Activity, which included any form of physical exertion such as walking up a hill/cycling or digging, was not significantly associated with the development of HT.

The use of fat/oil is another significant factor in this study. The odds of developing HT were higher among those who used fat/oil for cooking than among those who did not. Cooking with fats/oils increased the odds of HT development by 0.73.

This study did not find any significant association between HT, alcohol consumption, and smoking. This finding differs from studies that have indicated a significant association between HT and smoking, alcohol consumption, and fruit and vegetable consumption. This could be due to the indication of different risk factors for hypertension in Ndorwa West HSD among the Bakinga, since they accounted for the majority (86.5%). However, this is consistent with a study a previous study [23] done in Bangladeshi that did not find a significant association between smoking, fruit and vegetable consumption, and HT.

This community-based cross-sectional study had several limitations. This study established an association between HT and a number of risk factors and estimated their risks using odds ratios. As this was a cross-sectional study, it lacked the ability to establish causal relationship between observed associated risk factors and HT. HT was defined as the average of three BP readings taken at least 1 min apart and measured at the field level instead of the clinical setting level. The study also did not collect data on several other factors that are often associated with hypertension, such as a family history of HT, waist circumference, and cholesterol levels.

However, this study used a standard and pre-tested WHO STEPs questionnaire tailored to the study setting. The WHO STEPs questionnaire is widely used and, therefore, provides a reliable comparison of our findings with those of other studies.

## Conclusion

The prevalence of HT and pre-HT in this rapidly rural-urban transitioning population was high. The risk of CVDs is about 16 folds higher among pre-HT compared to no HT and doubles for every 10-mmHg increase in BP. Hence, the considerable risk and burden of HT and related CVDs that require a dire need to adopt strategies to prevent and control hypertension based on the identified associated risk factors in Ndorwa HSD.

## Recommendations

It is important to start awareness and prevention of hypertension campaigns through the Village Health team and other community health workers in the Ndorwa West HSD communicates based on identified modifiable risk factors, such as awareness of BP status, reduced salt intake, use of oil for cooking, and overweight and obesity status.

## Figures and Tables

**Figure 1:**
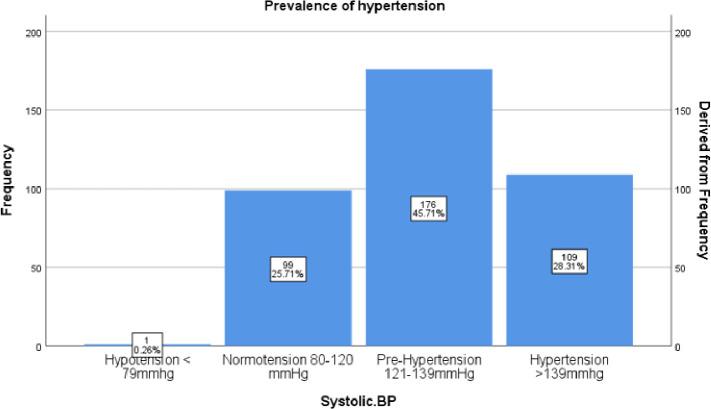
The prevalence of HT and pre-HT in Ndorwa HSD was 28.3% and 45.7% respectively

**Figure 2:**
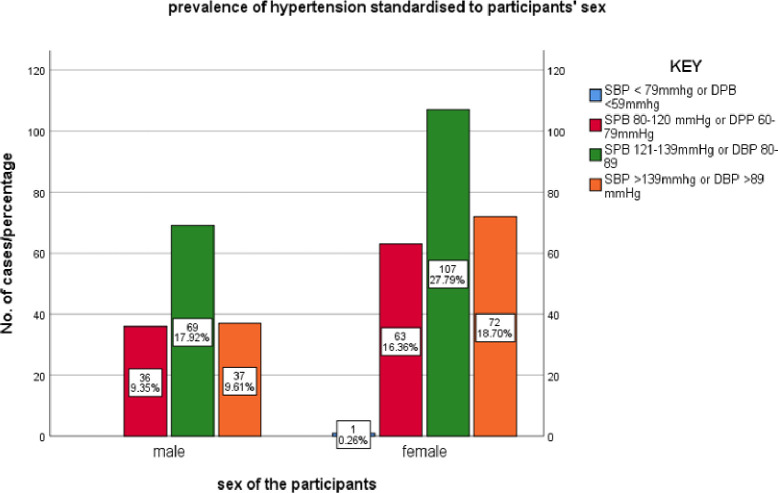
Prevalence of HT standardized to sex. Females had both higher pre-HT and HT prevalence compared to men.

**Figure 3:**
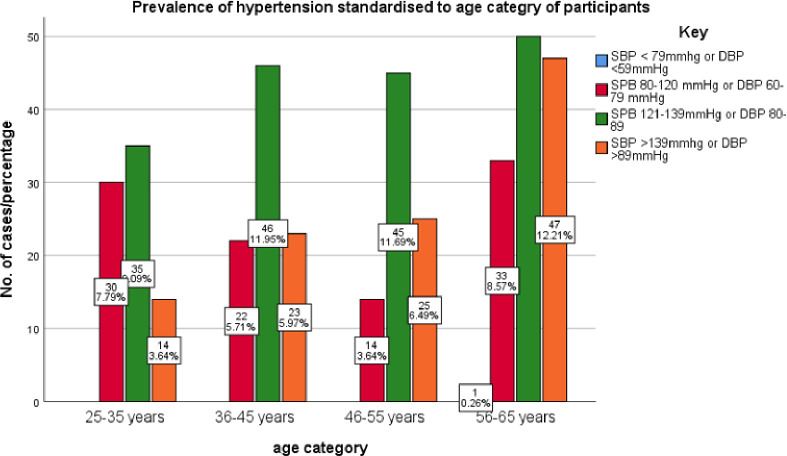
prevalence of HT standardized to age category. The prevalence of hypertension increased with age however it more rapidly after 55 years.

**Table 1:** Participants’ socio demographic and behavioral characteristics

Socio-demographic		
age category	Frequency	Percent
25–35	79	20.5
36–45	91	23.6
46–55	84	21.8
56–65	131	34.0
Total	385	100.0
Sex	Frequency	Percent
Male	142	36.9
Female	243	63.1
total	385	100
Tribe	Frequency	Valid Percent
Mukiga	333	86.5
munyankore	12	3.1
mufumbira/munyarwanda	40	10.4
**Total**	**385**	**100.0**
Marital status	Frequency	Valid Percent
single	25	6.6
married/cohabiting	236	62.1
separated/divorce/widowed	119	31.3
Total	380	100.0
Occupation	Frequency	Percent
un employed	221	58.0
self employed	113	29.7
Employed	47	12.3
Total	381	100.0
Level of education	Frequency	Percent
Informal	42	10.9
Primary	243	63.1
Secondary	63	16.4
Tertiary college/university	37	9.6
Total	385	100.0
Those who had ever had their BP measured	frequency	percent
yes	342	**89.5**
no	40	**10.5**
Total	382	**100.0**
BP awareness in the last 12 months	frequency	percent
yes	294	87.8
no	41	12.2
Total	335	100.0
BMI (kg/m^2^)	Frequency	Percent
BMI<18.5	3	0.8
BMI 18.5–24.9	173	44.9
BMI 25.0–29.9	157	40.8
BMI >30.0	52	13.5
Smoking	Frequency	Percent
Yes	51	13.2
No	334	86.8
Total	385	100.0
Alcohol consumption	Frequency	Percent
Yes	221	57.4
No	162	42.1
Total	385	100.0
**Fat/oil use for cooking**		
vegetable oil	130	33.8
butter/ghee/ margarine	92	23.9
none	163	42.3
Total	385	100.0
Activity(walk up hill, bicycling)	Frequency	Percent
Yes	151	39.2
No	234	60.8
Total	385	100.0
**Reduced Salt intake**		
Yes	345	91.8
No	31	8.2
Total	376	100.0

**Table 2:** Socio demographic and behavioral factors associated with hypertension

	Hypertension status			χ^2−^	p-value	Risk estimate. Odds Ratio, 95%CI	Remark
	
	No HTN	HTN	Total	**6.43**	**0.01**	**1.81, 1.14–2.87**	**Significant**

**Age category**							

45 years and below	133	37	170				

46 years and above	143	72	215				

Total	276	109	385				

**sex**				**0.56**	**0.45**	**1.19 (CI:0.75–1.90**	**Not significant**

Male	105	37	142				

Female	171	72	243				

Total	276	109	385				

**marital status**							

No partner	105	44	149	**0.02**	**0.65**	**0.57–1.43**	**Not significant**

Partner present	171	65	236				

Total	276	109	385				

**Overweight and obesity**				**6.05**	**0.01**	**1.77 (CI 1.12–2.80)**	**Significant**

BMI<24.9kg/m^2^	137	39	176				

BMI ≥25kg/m^2^	139	70	209				

Total	276	109	385				

**Level of education**				**8.28**	**0.41**	**risk not estimated**	**Significant**

informal	33	9	42				

primary	165	78	243				

secondary	45	18	63				

Tertiary	33	4	37				

Total	276	109	385				

**Employment**				**2.89**	**0.89**	**0.67 (CI:0.43–1.06)**	**Not significant**

un employed	151	70	221				

Employed	125	39	164				

Total	276	109	385				

**Smoking**				**0.963**	**0.810**	**1.33 (CI:0.68–2.64)**	**Not Significant**

yes	0	15	24				

no	1	84	152				

Total	1		176				

**Use of fat/oil for cooking**				**5.40**	**0.02**	**1.73 (CI: 1.09–2.75)**	**Significant**

no fat/oil use	127	36	163				

fat/oil use	149	73	222				

Total	276	109	385				

**Alcohol consumption**				**0.68**	**0.79**	**1.06(CI:0.69–1.66)**	**Not significant**

yes	161	62	223				

no	115	47	162				

Total	276	109	385				

**Reduced Salt intake**				**10.57**	**0.001**	**0.075(CI:0.01–0.55)**	**Significant**

yes	239	106	345				

no	30	1	31				

	269	107	376				

**Activity-walking up hill/cycling**				**1.21**	**0.27**	**1.29 (CI:0.82–2.06)**	**Not significant**

yes	110	37	147				

no	165	72	237				

	275	109	384				

**Awareness of BP status**				**6.48**	**0.01**	**0.53(CI: 0.32–0.87)**	**Significant**

yes	55	35	90				

No	221	74	295				

Total	276	109	385				

## Data Availability

The data is with the corresponding author and will be made available at a reasonable request
